# The Inhibition of Autophagy and Pyroptosis by an Ethanol Extract of *Nelumbo nucifera* Leaf Contributes to the Amelioration of Dexamethasone-Induced Muscle Atrophy

**DOI:** 10.3390/nu15040804

**Published:** 2023-02-04

**Authors:** Eunji Park, Hojung Choi, Cao-Sang Truong, Hee-Sook Jun

**Affiliations:** 1 Gachon Institute of Pharmaceutical Science, College of Pharmacy, Gachon University, 191 Hambakmoe-ro, Yeonsu-gu, Incheon 21936, Republic of Korea; 2 Lee Gil Ya Cancer and Diabetes Institute, Gachon University, 155 Gaetbeol-ro, Yeonsu-gu, Incheon 21999, Republic of Korea; 3 Gachon Medical Research Institute, Gil Hospital, 21 Namdong-daero774beon-gil, Namdong-gu, Incheon 21565, Republic of Korea

**Keywords:** muscle atrophy, nelumbo nucifera, pyroptosis, autophagy, protein degradation

## Abstract

Muscle atrophy is characterized by a decline in muscle mass and function. Excessive glucocorticoids in the body due to aging or drug treatment can promote muscle wasting. In this study, we investigated the preventive effect of *Nelumbo nucifera* leaf (NNL) ethanolic extract on muscle atrophy induced by dexamethasone (DEX), a synthetic glucocorticoid, in mice and its underlying mechanisms. The administration of NNL extract increased weight, cross-sectional area, and grip strength of quadriceps (QD) and gastrocnemius (GA) muscles in DEX-induced muscle atrophy in mice. The NNL extract administration decreased the expression of muscle atrophic factors, such as muscle RING-finger protein-1 and atrogin-1, and autophagy factors, such as Beclin-1, microtubule-associated protein 1A/1B-light chain 3 (LC3-I/II), and sequestosome 1 (p62/SQSTM1) in DEX-injected mice. DEX injection increased the protein expression levels of NOD-like receptor pyrin domain-containing protein 3 (NLRP3), cleaved-caspase-1, interleukin-1beta (IL-1β), and cleaved-gasdermin D (GSDMD), which were significantly reduced by NNL extract administration (500 mg/kg/day). In vitro studies using C2C12 myotubes also revealed that NNL extract treatment inhibited the DEX-induced increase in autophagy factors, pyroptosis-related factors, and NF-κB. Overall, the NNL extract prevented DEX-induced muscle atrophy by downregulating the ubiquitin–proteasome system, autophagy pathway, and GSDMD-mediated pyroptosis pathway, which are involved in muscle degradation.

## 1. Introduction

Skeletal muscle atrophy, which is defined as a reduction in muscle mass, occurs due to various causes, including denervation, inactivity due to injury, cancer, sepsis, kidney failure, heart failure, and excessive glucocorticoid conditions, such as Cushing’s syndrome [1,2]. According to the 2019 Global Burden of Disease study, approximately 1.71 billion people worldwide suffer from musculoskeletal disorders [3], and this number is expected to continue to increase in the coming decades. Therefore, therapeutic and prophylactic options for muscle atrophy are considered important for improving the quality of life. However, there are no FDA-approved drugs for the treatment of muscle atrophy [4]. Therefore, the development of new drugs to prevent or treat muscle atrophy is challenging.

An increase in circulating glucocorticoid hormone levels is associated with muscle atrophy in cachexia, sepsis, starvation, and aging [5,6]. Excess glucocorticoids cause muscle atrophy by increasing muscle protein degradation and decreasing muscle protein synthesis [7]. Dexamethasone (DEX), a class of synthetic glucocorticoids, is widely used to treat inflammation-related diseases. However, it is known to affect catalytic muscle atrophy when administered at high doses or for long periods [5].

Muscle degradation is caused by protein degradation through the ubiquitin–proteasome system (UPS) [8,9], autophagy [10,11], and programmed cell death, such as apoptosis and pyroptosis [12]. In contrast to apoptosis, which is characterized by immune silencing, pyroptosis causes the rupture of the cell membrane and the release of many cytokines and danger signaling molecules, which further activate the immune system and lead to an inflammatory response [12]. Since pyroptosis is initiated by caspase-1, a protease known as interleukin-1 beta (IL-1β)-converting enzyme [13], it is an inflammatory process of caspase-1-dependent programmed cell death. Gasdermin D (GSDMD) is also cleaved by activated caspase-1, and its N-terminal domain oligomerizes [14] and forms pores in the cell membrane to release inflammatory molecules, such as IL-1β, triggering pyroptotic cell death [15].

*Nelumbo nucifera* Gaertn (Nymphaeaceae) has been widely used for medicinal purposes in China, Japan, India, and Korea since prehistoric times [16]. *N. nucifera* contains several functional ingredients, including polyphenols, flavonoids such as myricetin, quercetin, and alkaloids. These physiologically active ingredients are associated with pharmacological actions such as anti-obesity, anti-cancer, anti-inflammatory, anti-aging, and anti-oxidative properties [17,18,19]. Muscle atrophy is accompanied by inflammation and oxidative stress [20,21], and a natural compound with anti-inflammatory or antioxidative properties may alleviate this condition [21,22,23]. A quercetin derivative, a component of the NNL, has anti-inflammatory properties and can attenuate DEX-induced muscle atrophy [22,24]. Furthermore, myricetin, which acts as an antioxidant, could also improve muscle strength [25]. Therefore, it is possible that an NNL extract may have beneficial effects on muscle atrophy. The water extract of the lotus leaf improves muscle strength and increases muscle mass [23], although the detailed mechanisms involved are not clearly understood. In this study, we sought to explore whether the ethanolic extract of NNL has beneficial effects on muscle atrophy and investigate the underlying mechanisms involved.

## 2. Materials and Methods

### 2.1. Nelumbo Nucifera Leaf Extract Preparation

In this study, a 70% (*v/v*) ethanol extract of dried NNL was used. Freeze-dried NNL ethanol-extracted powder (KOC201604-035) was purchased from KOC Biotech (Yuseong-gu, Daejeon, Republic of Korea). The dried ethanol extract was dissolved in Dimethylsulfoxide (DMSO) for in vivo experiments. For the in vitro experiments, the dried ethanol extracts were suspended in DMSO at concentrations of 50, 100, 200, and 500 mg/mL and stored at −20 °C.

### 2.2. High-Performance Liquid Chromatography (HPLC) Analysis

The HPLC analysis of the NNL ethanolic extract was performed using the HPLC Waters Arc HPLC System at KOC Biotech (Daejeon, Republic of Korea). HPLC-grade solvents, such as methanol, acetonitrile, and acetic acid (JT Baker, Avantor Materials, PA, USA), were used. Sample and standard solutions were prepared by dissolving NNL ethanolic extract and standards in methanol three times (30 min total) at 10-min intervals using ultrasonic waves. Compounds were separated using an XBridgeTM C18 column (5 μm, 4.6 × 250 mm). The column temperature was maintained at 30 °C and the flow rate was set at 0.6 mL/min. The injection volume was 10 μL and the absorbance was measured at a wavelength of 350 nm. Mobile phase A, 0.2% acetic acid in water, and mobile phase B, acetonitrile, were employed in the analyses with the following gradients: 0–35 min 88% A, 0–35 min 12% B: 36–55 min 90% A, 36–55 min 10% B: 56–70 min 30% A, 56–70 min 70% B: 71–100 min 88% A, 71–100 min 12% B.

### 2.3. Animals

Seven-week-old C57BL/6N male mice were purchased from Orient Bio (Seongnam-si, Gyeonggi-do, Republic of Korea) and allowed 1 week of adaptation before the study. The mice were maintained at a temperature of 23 ± 1 °C with a 12/12 h light/dark cycle and were provided free access to water and food. All animal experiments were conducted in accordance with the ethical requirements of the Experimental Animal Research Center, College of Pharmacy, Gachon University, Seongnam-si, Republic of Korea. The experimental protocol was approved by the Gachon University Institutional Animal Care Committee (GIACUC-R2019038).

### 2.4. DEX-Induced Muscle Atrophy in Mice and Administration of NNL Extract

C57BL/6N male mice (8 weeks old) were randomly divided into the following four groups (*n* = 11 in each group): normal control (CON), Dexamethasone (DEX) group (20 mg/kg body weight), DEX + NNL extract 200 mg/kg (DEX + NNL 200) group, and DEX + NNL extract 500 mg/kg (DEX + NNL 500) group. Muscle atrophy was induced by intraperitoneal (i.p.) injection of DEX (20 mg/kg/day for 13 days; D1756, Sigma-Aldrich, St. Louis, MO, USA) in C57BL/6N male mice. NNL extract (200 or 500 mg/kg/day) was orally administered for 15 days from 2 days before DEX injection. The DEX and NNL were dissolved in DMSO:10% Kolliphor^®^ HS 15 (1:9, *v/v*; 42966, Sigma-Aldrich). The mice in the control group received the vehicle only. Bodyweight and food intake were monitored daily during the experiment. The mice were sacrificed, and five types of muscles were isolated and weighed: quadriceps femoris (QD), gastrocnemius (GA), tibialis anterior (TA), soleus (SOL), and extensor digitorum longus (EDL). Tissues from sacrificed mice were stored at -80 °C or fixed.

### 2.5. Measurement of Grip Strength

A grip strength test was performed 14 days after NNL-extract administration using a grip strength meter (BIO-G53, BIOSEB, Pinellas Park, FL, USA). To assess muscle strength, the grip strength was measured as previously described [21]. The grip strength was calculated as force divided by the final body weight (N/g).

### 2.6. Hematoxylin and Eosin Staining 

The muscle tissues were fixed in 10% neutral buffered formalin, embedded in paraffin, and cut into 2.5 µm thick sections. For staining, sections were deparaffinized by incubation in xylene, hydrated in a series of ethanol concentrations (100, 90, 70, and 50%), washed in distilled water, stained with hematoxylin (MA0101010, BBC Biochemical, McKinney, TX, USA), and then stained with eosin (MA0101015, BBC Biochemical) (H&E). After washing, the sections were rapidly dehydrated in ethanol (50, 70, 90, and 100%). Finally, the sections were washed with xylene and mounted. The stained sections were photographed using a microscope (Eclipse 80i, Nikon Co., Tokyo, Japan) and NIS-element AR 4.00.00 software.

### 2.7. TUNEL Staining 

The muscle tissue sections were stained using TUNEL Apoptosis Detection Kit (KTA2010, Abbkine Scientific, Wuhan, China) according to the manufacturer’s protocol. The stained sections were photographed using a laser scanning confocal microscope (Eclipse A1 Plus, Nikon Co., Otawara, Japan). The cell nuclei were counterstained with DAPI and cell nuclei showing green fluorescence were defined as pyroptotic cells. The images were analyzed in the five randomly selected areas.

### 2.8. Cell Culture

The C2C12 cells (CRL-1772, ATCC^®^, Manassas, VA, USA) were incubated at 37 °C in 5% CO_2_. The cells were cultured in Dulbecco’s Modified Eagle Medium (DMEM; LM001-05, Welgene, Gyeongsangbuk-do, Republic of Korea) containing 10% fetal bovine serum (S001-07, Welgene) and 1% ZellShield™ (#13-0150, Minerva-Biolabs, Berlin, Germany). To differentiate C2C12 myoblasts into C2C12 myotubes, the cells were seeded at 2.5 × 105 cells/well in a 6-well plate and cultured at full density for 2 days. The medium was then replaced with a differentiation medium containing 2% horse serum (#16050-122, Thermo Fisher Scientific, Waltham, MA, USA) and 1% ZellShield™ and changed daily for 5 days. To determine the effect of the NNL extract on the DEX-induced cell damage, the differentiated C2C12 myotubes were treated with 100 μM DEX and 50, 100, and 200 μg/mL NNL extract for 24 h.

### 2.9. CCK-8 Assay

The CCK-8 assay was performed to check the viability of the C2C12 cells after treatment with the NNL extract. The C2C12 cells were seeded in a 96-well plate at a density of 5 ×  10^3^ cells/well. The cells were treated with different concentrations of NNL extract (diluted with DMSO 25, 50, 100, 200, or 500 µg/mL) and the control group was treated with the vehicle only. The D-Plus™ CCK cell viability assay kit (CCK-3000, Dongin LS, Seoul, Republic of Korea) was added to each well and the absorbance was measured at 450 nm after 2 h of incubation.

### 2.10. Western Blotting (Immunoblotting)

The QD muscle tissue (20 mg), or C2C12 myotubes, were lysed in 200 μL (for tissues) or 50 μL (for cells) of a mammalian protein extract buffer (#78501, Thermo Fisher Scientific, Waltham, MA, USA) containing a protease inhibitor Cocktail (P8340, Sigma-Aldrich), a phosphatase inhibitor Cocktail 2 (P5726, Sigma-Aldrich), and a phosphatase inhibitor Cocktail 3 (P0044, Sigma-Aldrich). Proteins were electrophoresed on SDS-polyacrylamide gels and then transferred to NC membranes (GE Healthcare Life Science, Buckinghamshire, UK). After blocking with 5% skim milk for 1 h, the membrane was incubated with the primary antibodies at a 1:1,000 dilution for the anti-MuRF1 (ab172479, Abcam, Cambridge, MA, USA), anti-MAFbx/Atrogin-1 (ab74023, Abcam), anti-LC3-I/II (#12741, Cell Signaling, Danvers, MA, USA), anti-SQSTM/p62 (#5114, Cell Signaling), anti-Beclin-1 (#3495, Cell Signaling), anti-Caspase-1 (ALX-210-804-C100, Enzo Biochem, Farmingdale, NY, USA), anti-IL-1β (SC-52012, Santa Cruz, CA, USA), anti-NLPR3/NALP3 (NBP2-12446, Novus Biologicals, Centennial, CO, USA), anti-GSDMDC1 (NBP2-33422, Novus Biologicals), anti-p70S6 Kinase (#9202, Cell Signaling), anti-p-p70S6 Kinase (Thr389) (#9205, Cell Signaling), anti-p44/42 MAPK (Erk1/2) (#9102, Cell Signaling) or anti-p-p44/42 MAPK (Erk1/2) (Thr202/Tyr204) (#9101, Cell Signaling), anti-NF-κB p65 (C22B4) (#4764, Cell Signaling), anti-p-NF-κB p65 (Ser 536) (#3033, Cell Signaling), anti-IκBα (L35A5) (#4814, Cell Signaling), and anti-p-IκBα (Ser32/36) (5A5) (#9246, Cell Signaling), or at a 1:5000 dilution for the anti-GAPDH (SC-32233, Santa Cruz, CA, USA). After overnight incubation at 4 °C, the nitrocellulose membrane was washed three times with 1 × TBS-T for 10 min and incubated with horseradish peroxidase-conjugated goat anti-mouse IgG (A90-116P, Bethyl Laboratories, Montgomery, TX, USA) or goat anti-rabbit IgG (A120-101P, Bethyl Laboratories) secondary antibodies. Signals were detected using a Chemidoc™ XRS + system with Image Lab™ software (Bio-Rad, Hercules, CA, USA) using an Immobilon Western Chemiluminescent HRP Substrate ECL Detection Kit (WBKLS0500, Millipore Burlington, MA, USA). Band intensity was quantified using ImageJ software (National Institutes of Health, Bethesda, MD, USA).

### 2.11. Statistical Analyses

Data are presented as mean ± standard error of mean (S.E.M). The statistical analysis was performed by a one-way unpaired parametric analysis of variance (ANOVA) using the GraphPad Prism 7 software. The statistical significance was set at *p* < 0.05.

## 3. Results

### 3.1. HPLC Analysis of NNL Ethanolic Extracts

**Figure 1:**
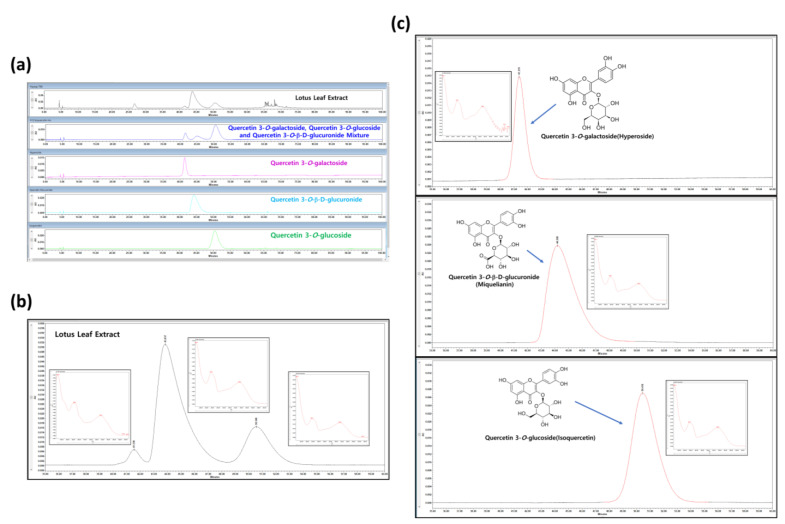
The HPLC analysis of NNL ethanolic extracts. The NNL ethanolic extract and three standards (Quercetin 3-O-galactoside (hyperoside), Quercetin 3-O-β-D-glucuronide (miquelianin), and Quercetin 3-O-glucoside (isoquercetin)) were used in the HPLC analyses. (**a**) HPLC chromatograms comparing NNL extract and standards. (**b**) Photodiode Array (PDA) and HPLC chromatograms of NNL extract. (**c**) Photodiode Array (PDA) and HPLC chromatograms of reference standards.

Comparative HPLC chromatograms of NNL ethanolic extract were carried out. Absorbance was measured at 350 nm based on previously obtained information [19]. Strong absorbance peaks were obtained for each of the three compounds. The respective peaks within the NNL ethanolic extract eluted at 41.3, 43.8, and 50.5 min (Figure 1a,b). The HPLC analysis was performed by selecting Quercetin 3-O-galactoside (hyperoside), Quercetin 3-O-β-D-glucuronide (miquelianin), and Quercetin 3-O-glucoside (isoquercetin), based on the previously known standard components of NNL extract [18,19]. The peaks of the standard were eluted at 41.3, 44.1, and 50.4 min, respectively, confirming that Quercetin 3-O-galactoside (hyperoside), Quercetin 3-O-β-D-glucuronide (miquelianin), and Quercetin 3-O-glucoside (isoquercetin) were the main components of the NNL ethanolic extract that we used for the experiments (Figure 1b,c).

### 3.2. The NNL Extract Administration Increased Body and Muscle Weights in DEX-Induced Muscle Atrophy in Mice

**Figure 2:**
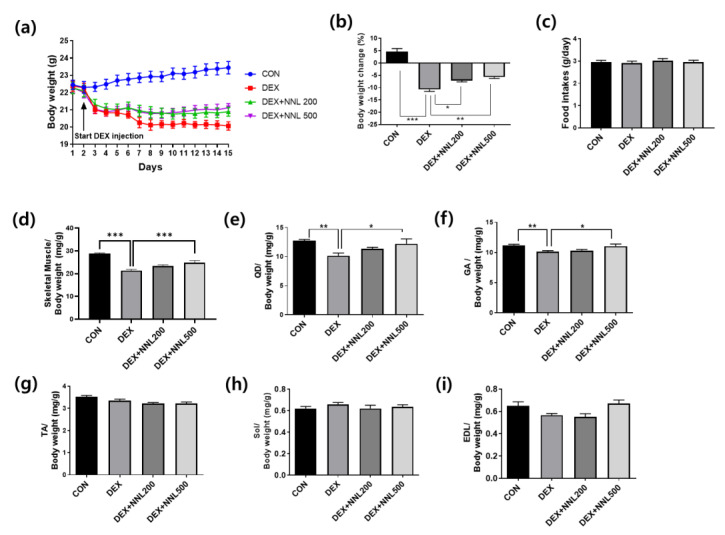
The NNL extract administration increased body and muscle weights in DEX-induced muscle atrophy in mice. The NNL extract (200 or 500 mg/kg/day) was orally administered to C57BL/6 male mice (8 weeks old) once a day for 15 days. Two days after NNL extract administration, DEX (20 mg/kg/day) was intraperitoneally injected once a day for 13 days. (**a**) Changes in body weight during the experimental periods. (**b**) The calculation of body weight changes compared to the body weight in day 0. (**c**) Food intake during the experimental period. (**d**) The measurement of total skeletal muscle weights. (**e**) Quadriceps (QD) muscle weight. (**f**) Gastrocnemius (GA) muscle weight. (**g**) Tibialis anterior (TA) muscle weight. (**h**) Soleus (SOL) muscle weight. (**i**) Extensor digitorum longus (EDL) muscle weight. All data are shown as mean ±S.E.M., *n* = 11/group; * *p* < 0.05, ** *p* < 0.01, *** *p* < 0.001.

To evaluate the effects of NNL ethanol extracts on DEX-induced muscle atrophy, 8-week-old C57BL/6N male mice were orally administered an NNL extract 2 days before DEX administration for a total of 15 days. As shown in Figure 2a,b, the NNL-extract administration (both DEX + NNL 200 and DEX + NNL 500 groups) inhibited the body weight loss induced by the DEX injection. No changes in food intake were found among the groups (Figure 2c). In particular, total muscle weight and the weight of QD and GA muscles were significantly increased by administration of 500 mg/kg NNL extract in DEX-induced muscle atrophy in mice (Figure 2d–f). The weights of the TA, SOL, and EDL did not show significant changes (Figure 2g–i).

### 3.3. NNL Extract Administration Increased Grip Strength and Muscle Myofiber Size in DEX-Induced Muscle Atrophy in Mice

**Figure 3:**
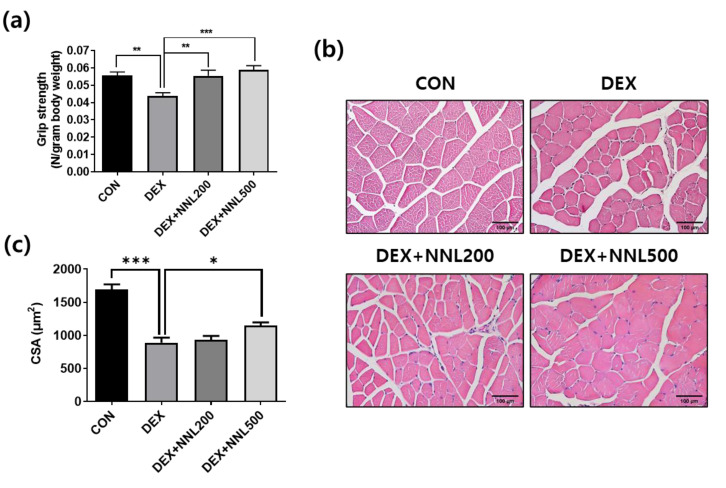
The NNL extract administration increased grip strength and muscle myofiber size in DEX-induced muscle atrophy in mice. (**a**) A grip strength test was performed 14 days after NNL extract administration. *n =* 11/group. (**b**) The QD muscle tissue sections were stained with H&E and observed under a microscope. Representative images of H&E-stained sections (original magnification, 100×). (**c**) Cross-sectional area (CSA) of QD muscle fiber was measured using the ImageJ program and mean CSA is shown. *n* = 4/group. Data are shown as mean ± S.E.M., * *p* < 0.05, ** *p* < 0.01, *** *p* < 0.001.

To determine whether the NNL extract administration improved muscle function, we measured grip strength 14 days after its administration. Grip strength was significantly decreased in the DEX group and significantly increased in both the DEX+NNL 200 and DEX + NNL 500 groups (Figure 3a). The QD muscle fiber size analysis showed that the cross-sectional area (CSA) was reduced by approximately 50% in the DEX group compared to that in the CON group. The DEX + NNL 500 group showed a significantly increased CSA of the QD muscle fibers compared to the DEX group (Figure 3b,c). The size of muscle fibers and grip strength were further increased, but not significantly, in the DEX + NNL 500 group compared to those in the DEX + NNL 200 group (Figure 3a–c).

### 3.4. The NNL-Extract Administration Decreased the Expression of Muscle Degradation Factors and Increased the Expression of Muscle Synthesis Factors in DEX-Induced Muscle Atrophy in Mice

**Figure 4:**
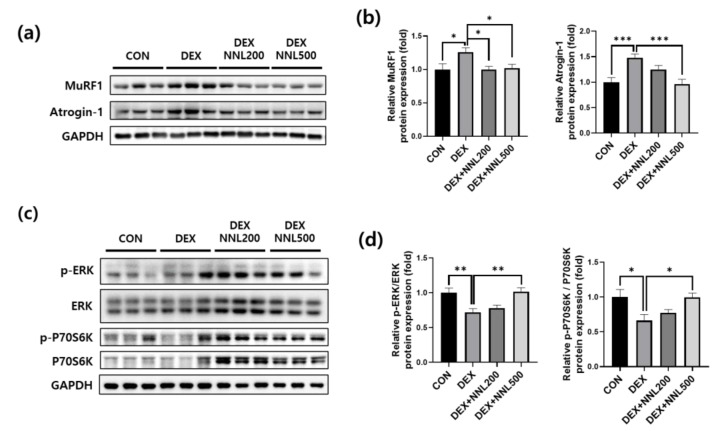
The NNL extract decreased the expression of muscle degradation factors and increased the expression of muscle synthesis factors in DEX-induced muscle atrophy in mice. (**a,b**) The protein levels of MuRF1 and atrogin-1 in the QD muscle were analyzed by immunoblotting, quantified using ImageJ software, and normalized to those of GAPDH. (**c,d**) The protein levels of phosphorylated ERK and P70S6K in the QD muscle were analyzed by immunoblotting, quantified using ImageJ software, and normalized to ERK and P70S6K, respectively. Representative images (**a,c**) and quantitative analysis bar graphs (**b,d**) are shown. Data are shown as mean ±S.E.M., *n* = 6/group; * *p* < 0.05, ** *p* < 0.01, *** *p* < 0.001.

Muscle RING-finger protein-1 (MuRF1) and atrogin-1 are E3 ligases involved in ubiquitin–proteasome-mediated protein degradation [26]. We examined the protein expression of MuRF1 and atrogin-1 in QD muscle and found that their expression was significantly increased by DEX injection, but significantly decreased by the NNL extract administration (Figure 4a,b). The ERK and p70S6K signaling molecules are well-known regulators of protein synthesis [27,28]. To evaluate the effects of the NNL extract on muscle protein synthesis, we investigated the expression of phosphorylated ERK and p70S6K. Phosphorylated ERK and P70S6K were significantly reduced in the DEX group compared to those in the CON group; however, administration of 500 mg/kg NNL extract significantly inhibited these reductions induced by the DEX injection (Figure 4c,d).

### 3.5. The NNL-Extract Administration Inhibited the Expression of Autophagy-Related Factors in DEX-Induced Muscle Atrophy in Mice

**Figure 5:**
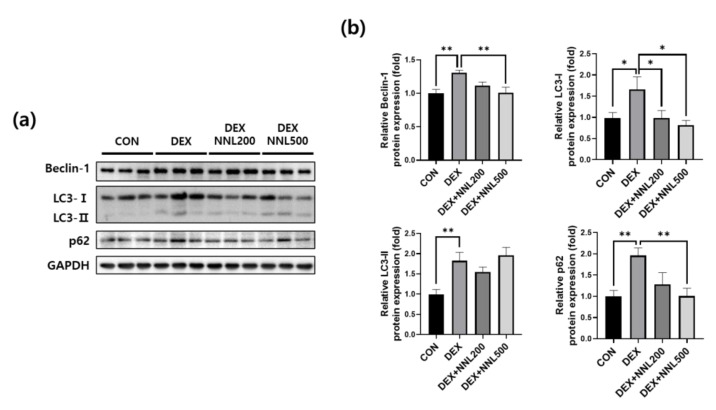
The NNL-extract administration inhibited the expression of autophagy-related factors in DEX-induced muscle atrophy in mice. The protein expression levels of Beclin-1, LC3-I/II, or p62 in the QD muscle tissue were analyzed by immunoblotting. GAPDH was used as an internal control. (**a**) The representative images are shown. (**b**) Beclin-1, LC3-I/II, or p62 immunoblots were quantified in Figure 5a using ImageJ software and normalized to GAPDH. Data are shown as the mean ±S.E.M., *n* = 6/group; * *p* < 0.05, ** *p* < 0.01.

Increased autophagy leads to muscle loss by mediating protein degradation in cooperation with the ubiquitin–proteasome system [29]. Beclin-1 and LC3 are autophagosome components, and p62 interacts with ubiquitin marker proteins and autophagosomes [30]. To investigate whether the NNL extract administration affects the expression of autophagy-related factors, we evaluated the protein expression levels of Beclin-1, microtubule-associated protein 1A/1B-light chain 3-I/II (LC3-I/II), and p62/SQSTM1 (p62). We found that the protein levels of Beclin-1, LC3-I/II, and p62 were significantly increased in the QD muscle by the DEX injection. However, administration of 500 mg/kg NNL extract significantly decreased the DEX-induced increases in Beclin-1, LC3-I, and p62, but not in LC3-II (Figure 5a,b). These results indicate that the NNL extract may inhibit autophagy in muscles of DEX-injected mice.

### 3.6. The NNL Extract Administration Inhibited Pyroptosis in DEX-Induced Muscle Atrophy in Mice

**Figure 6:**
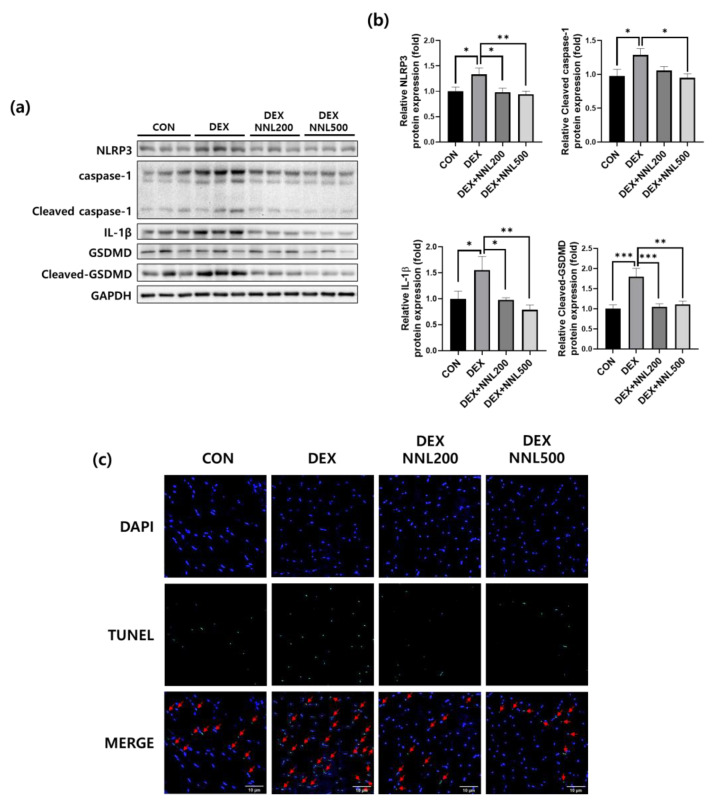
The NNL extract administration inhibited pyroptosis in DEX-induced muscle atrophy in mice. The protein expression levels of NLRP3, Caspase-1, IL-1β, or GSDMD in the QD muscle were analyzed by immunoblotting. GAPDH was used as an internal control. (**a**) Representative images are shown. (**b**) NLRP3, cleaved caspase-1, IL-1β, cleaved-GSDMD immunoblots were quantified in Figure 6a using ImageJ software and normalized to GAPDH. Data are shown as mean ±S.E.M., *n* = 6/group; * *p* < 0.05, ** *p* < 0.01, *** *p* < 0.001. (**c**) TUNEL staining of the QD muscle sections. Representative images are shown (original magnification, 400×; *n* = 5/group). DAPI (blue) and TUNEL-positive cells (green). The red arrows indicate TUNEL-positive cells.

Increased inflammation is accompanied by cellular pyroptosis, which leads to significant muscle cell loss and adverse remodeling in diabetic myopathy [31]. In addition, pyroptosis was recently reported to be involved in muscle damage during DEX-induced muscle atrophy [32]. To investigate the effect of the NNL extract on pyroptosis in DEX-induced muscle atrophy, we investigated the expression of pyroptosis-related factors in the QD muscle. The protein expression levels of NOD-like receptor pyrin domain-containing protein 3 (NLRP3), cleaved caspase-1, and mature IL-1β were significantly increased by DEX injection, whereas the administration of NNL extracts significantly decreased these (Figure 6a,b). In addition, the expression level of GSDMD, a key pyroptosis factor, was significantly decreased in the NNL treatment group compared to that in the DEX group (Figure 6a,b). These results suggest that the NNL extract could inhibit pyroptosis, contributing to the prevention of muscle atrophy in DEX-injected mice. Pyroptosis is characterized by cell expansion, terminal deoxynucleotidyl transferase dUTP nick-end labeling (TUNEL) staining, and pyroptotic DNA fragmentation [33,34,35]. Therefore, we performed a TUNEL assay on the QD muscle sections. The TUNEL-stained cells were increased in DEX-induced muscle atrophy in mice compared to those in the control mice (Figure 6c). However, this increase was inhibited by the NNL extract at doses of both 200 mg/kg and 500 mg/kg (Figure 6c).

### 3.7. The NNL Extract Decreased the Expression of Muscle Degradation and Autophagy Factors in DEX-Treated C2C12 Myotubes

**Figure 7:**
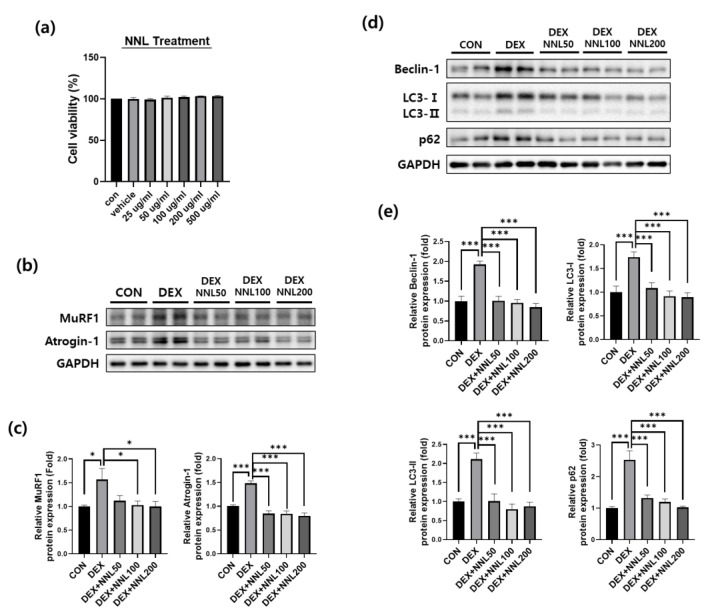
The NNL extract decreased the expression of the muscle degradation and autophagy factors in DEX-treated C2C12 myotubes. (**a**) C2C12 myoblasts were treated with NNL extract (25, 50, 100, 200, or 500 μg/mL) for 24 h and cell viability was analyzed by CCK8 assay. (**b**-**e**) Differentiated C2C12 myotubes were treated with 50, 100, or 200 μg/mL NNL extract for 24 h in the presence of DEX (100 μM). The protein expression levels of MuRF1 and atrogin-1 (**b,c**), Beclin-1, LC3-I/II, and p62 (**d,e**) were analyzed by immunoblotting, quantified using ImageJ software, and normalized to GAPDH. The representative images (**b,d**) and quantitative analysis bar graphs (**c,e**) are shown. Data are shown as the mean ±S.E.M., *n* = 3/group; * *p* < 0.05, *** *p* < 0.001.

Having identified the protective effects of the NNL extract on DEX-induced muscle atrophy in mice, we validated these findings using differentiated C2C12 myotubes. First, we investigated the cytotoxicity of the NNL extract in C2C12 cells. The cells were treated with various concentrations of NNL extracts (25, 50, 100, 200, or 500 µg/mL) and incubated for 24 h, and cell viability was analyzed using the D-Plus™ CCK-8 cell viability assay kit. We observed that cell viability did not change at any of the tested concentrations (Figure 7a). Therefore, we used 50, 100, or 200 μg/mL of the NNL extract for in vitro experiments. To investigate the effects of the NNL extract on the expression of muscle degradation factors, the differentiated C2C12 myotubes were treated with different concentrations of NNL extract (50, 100, or 200 μg/mL) for 24 h in the presence of DEX. The DEX significantly increased the expression of MuRF1 and atrogin-1 proteins in C2C12 myotubes. However, the NNL extract significantly inhibited the DEX-induced increase in atrogin-1 and MuRF1 expression. We examined the expression of autophagy-related factors and found that the expression of Beclin-1, LC3-I/II, and p62 was significantly increased by the DEX treatment; however, the NNL extract significantly inhibited this increase in a dose-dependent manner (Figure 7d,e).

### 3.8. The NNL Extract Decreased the Expression of Pyroptosis Factors in DEX-Treated C2C12 Myotubes

**Figure 8:**
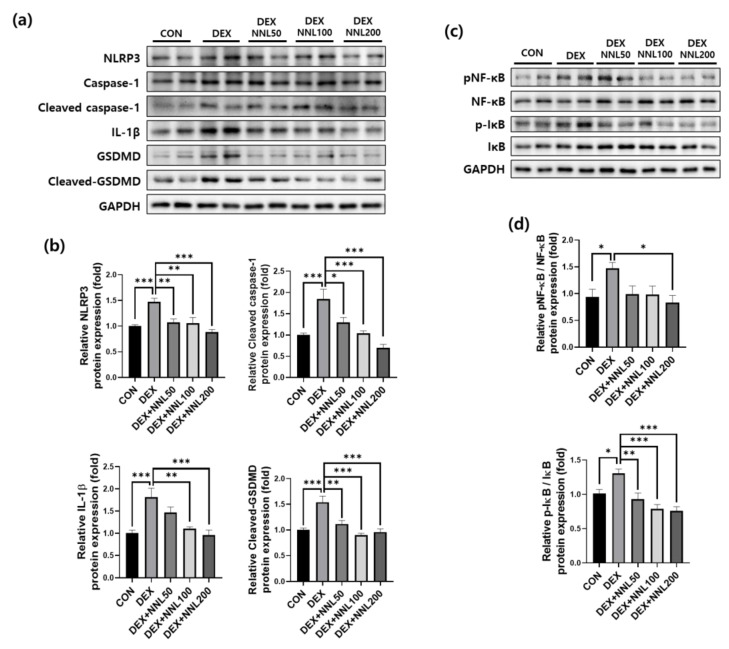
The NNL extract decreased the expression of pyroptosis factors in DEX-treated C2C12 myotubes. Differentiated C2C12 myotubes were treated with 50, 100, and 200 μg/mL NNL extract for 24 h in the presence of DEX (100 μM). (**a,b**) The protein expression levels of NLRP3, cleaved caspase-1, IL-1β, and cleaved-GSDMD were analyzed by immunoblotting, quantified using ImageJ software, and normalized to GAPDH. (**c,d**) The protein expression levels of pNF-κB and p-IκB were analyzed by immunoblotting, quantified using ImageJ software, and normalized to NF-κB and IκB, respectively. Representative images (**a,c**) and quantitative analysis bar graphs (**b,d**) are shown. Data are shown as the mean ±S.E.M., *n* = 6/group; * *p* < 0.05, ** *p* < 0.01, *** *p* < 0.001.

To investigate the direct effects of the NNL extract on the expression of pyroptosis-related factors, we treated differentiated C2C12 myotubes with NNL extracts and evaluated the protein expression of NLRP3, cleaved caspase-1, IL-1β, and cleaved-GSDMD. The expression of these proteins was significantly increased by the DEX treatment. Treatment with the NNL extract decreased the expression of pyroptosis factors in a concentration-dependent manner (Figure 8a,b). In addition, it has been reported that NF-κB and IκB act as major molecules regulating pyroptosis [36]. Therefore, we examined the activation of NF-κB and IκB using immunoblotting. The phosphorylation levels of these proteins were significantly increased by the DEX treatment and decreased with the NNL-extract treatment (Figure 8c,d). 

## 4. Discussion

The skeletal muscle constitutes approximately 40% of the total body mass and is important for movement, energy metabolism, and respiration. Skeletal muscle mass is maintained through a dynamic balance between protein synthesis and degradation. Therefore, if protein degradation is excessive compared to protein synthesis, the muscle mass and function are decreased, resulting in skeletal muscle atrophy [37]. Skeletal muscle atrophy is a serious complication that can occur in a variety of conditions, including diabetes, cancer, heart failure, bed rest, and nerve injury [38,39,40] and degrades the quality of life and increases morbidity and mortality [41]. Exercise is the typical treatment for muscle atrophy. However, in cases of the inability to exercise due to weakness, treatment with drugs is needed, and many agents, including natural components, have been developed for the treatment of muscle atrophy [38]; however, no drug has yet been clinically approved. 

*N. nucifera* belongs to the Nymphaeaceae family and is cultivated as a crop mainly in eastern Asia and India. All parts of *N. nucifera* have been used for various medicinal purposes in various systems of medicine. In this study, we investigated the detailed molecular mechanisms underlying the beneficial effects of *N. nucifera* leaf ethanol extract and found that the NNL extract ameliorated muscle atrophy by inhibiting the autophagy and pyroptosis pathways. 

We initially investigated the effect of the NNL ethanolic extract on muscle atrophy using DEX-induced muscle atrophic C57BL/6 mice. As expected, the NNL extract significantly increased body weight and skeletal muscle weight when compared with the DEX group. In particular, the increase in the QD and GA muscles was most significant when the NNL extract was administered (Figure 2e). Grip strength was improved and CSA was also increased by the NNL-extract administration in DEX-induced muscle atrophy in mice (Figure 3). These results suggest that the administration of the NNL ethanol extract inhibits the decrease in muscle mass and function caused by the DEX injection. 

Muscles are maintained through the balance of synthesis and degradation, and when the wasting ratio is higher than that of muscle synthesis, muscle atrophy occurs [10]. NNL extract administration significantly increased the expression of protein synthesis markers, such as ERK and p70S6K phosphorylation (Figure 4), which are known to be decreased by DEX [42,43]. DEX inhibits p70S6k phosphorylation [27,28], and it has been reported that the activation of ERK has a similar effect to Akt phosphorylation on protein synthesis [44]. These results suggest that the increase in muscle protein synthesis through the increase in phosphorylated ERK and p70S6K pathways may contribute to the increase in muscle mass induced by the NNL extract.

The UPS and the autophagy–lysosomal pathways are the two main pathways involved in muscle degradation [45]. The expression of the E3 ubiquitin ligases atrogin-1 and MuRF1 is highly upregulated in muscles undergoing atrophy [46]. DEX increases the protein expression levels of these factors, leading to muscle wasting [42]. We showed that DEX injection induces muscle atrophy in C57BL/6 mice through the upregulation of E3 ubiquitin ligase protein levels (Figure 4). The administration of NNL extract exerted a protective effect by inhibiting the expression of these factors in DEX-induced muscle atrophy in mice. 

Autophagy is an autolytic process that removes misfolded or aggregated proteins and organelles, eliminates intracellular pathogens, and promotes the proteolysis of cytoplasmic components in lysosomes [47]. In addition, autophagy plays an important role in mediating protein degradation through the ubiquitin–proteasome system [1]. It is well established that Beclin-1, LC3-I/II, and p62 are proteins involved in the formation of autophagosomes [48,49]. Beclin-1 binds to LC3-I during autophagosome formation and is converted to its membrane-bound form (LC3-II). LC3 binds to the adapter protein p62, which promotes the degradation of ubiquitinated proteins in the lysosomes. [50]. According to previous reports, muscle wasting is improved through autophagy during muscle atrophy [51], but excessive autophagy increases muscle loss [10]. Considering the evidence that DEX-induced autophagy mediates muscle atrophy [11], we investigated whether the NNL extract reduced the expression of Beclin-1, LC3-I/II, and p62. We found that the expression of these proteins was significantly decreased both in vivo and in vitro (Figure 5 and Figure 7). These data suggest that the NNL extract could prevent muscle loss by inhibiting autophagy in DEX-induced muscle atrophic mice. In our study, the expression level of LC3-Ⅱ did not change in the NNL-administered groups compared with that in the DEX group in vivo (Figure 5). The exact reason for this phenomenon is unknown. Giménez-Xavier et al. reported that LC3 can bind to phospholipids and intracellular membranes, but these structures may not change into autophagic vacuoles [51], indicating that the conversion of LC3-I to LC3-II is not essential for complete autophagy. 

Pyroptosis can cause inflammation that is activated by extracellular or intracellular stimuli [52] and is associated with various diseases, such as cardiovascular disease [53], Alzheimer’s disease [54], atherosclerosis [55], and muscle atrophy [56]. A recent study showed that pyroptosis occurs during dexamethasone-induced muscle atrophy [57]. NLRP3 inflammasome stimulates the activation of caspase-1, which plays a central role in cellular immunity as an inflammatory response initiator. Activated caspase-1 induces IL-18 and IL-1β [58]. Activated (cleaved) caspase-1 also cleaves GSDMD, activating the GSDMD-N domain to form membrane pores. Therefore, caspase-1 activation can lead to rapid cell death and the production of activated inflammatory cytokines [59]. NLRP3-dependent caspase-1 activity has been reported to increase with age in the skeletal muscles of mice [60]. In addition, high concentrations of DEX activate the GSDMD pathway-mediated pyroptosis by promoting an inflammatory response in mice [57,61]. In our study, NLRP3 expression was significantly reduced by the NNL-extract administration in DEX-induced atrophy in mice (Figure 6). Similarly, the NNL extract significantly decreased DEX-induced NLRP3 expression in C2C12 myotubes (Figure 8). Consistently, the protein expression of pyroptosis cascade markers, such as cleaved caspase-1, IL-1β, and cleaved-GSDMD, was also reduced in the NNL extract-administered groups, both in muscle tissue and C2C12 myotubes. These results suggest that the NNL extract can prevent pyroptosis, thereby preventing muscle degradation. Consistent with our results, another report showed that the reduction of pyroptosis factors contributed to the amelioration of muscle atrophy induced by DEX [61]. Additionally, it was recently reported that the administration of bone morphogenetic protein 7 (BMP7) attenuated diabetic myopathy by inhibiting pyroptosis and inflammation in mice [31].

NF-κB is an important signaling pathway associated with the loss of skeletal muscle mass [62]. In addition, a recent study reported that NF-κB deletion prevented muscle loss induced by hindlimb unloading [63]. NF-κB is known to regulate the inflammatory response, and NF-κB activation induces the expression of various pro-inflammatory genes and participates in the regulation of inflammasomes [64]. NF-κB is a central mediator of NLRP3 inflammasome activation and induces the transcriptional expression of NLRP3 and pro-IL-1β in response to various cytokines [65]. Since NF-κB induces the expression of NLRP3 and ultimately induces pyroptosis, we examined the protein expression levels of p-NF-κB and p-IκB. It was confirmed that the activation of NF-κB and IκB increased in the DEX group, and this activation was significantly decreased in the NNL extract treatment group. Thus, it was confirmed that NF-κB activation can induce pyroptosis to cause muscle atrophy, and it was found that NNL extract inhibits muscle degradation by blocking the NFκB-GSDMD pathway.

## 5. Conclusions

In conclusion, our data showed that the NNL extract alleviates muscle loss and increases muscle strength in DEX-induced muscle atrophy via inhibition of Beclin-1/LC3-mediated autophagy, the GSDMD-mediated pyroptosis pathway, and inhibition of ubiquitin-mediated muscle degradation. The pathway described herein could be a potential mechanism for the prevention of muscle dysfunction.

## Data Availability

The data used to support the findings of this study are available from the corresponding author upon request.
